# Community-Based Health Insurance Increased Health Care Utilization and Reduced Mortality in Children Under-5, Around Bwindi Community Hospital, Uganda Between 2015 and 2017

**DOI:** 10.3389/fpubh.2018.00281

**Published:** 2018-10-09

**Authors:** Nahabwe Haven, Andrew E. Dobson, Kuule Yusuf, Scott Kellermann, Birungi Mutahunga, Alex G. Stewart, Ewan Wilkinson

**Affiliations:** ^1^Church of Uganda Bwindi Community Hospital, Kinkizi Diocese, Kanungu, Uganda; ^2^College of Life and Environmental Science, University of Exeter, Exeter, United Kingdom; ^3^Institute of Medicine, University of Chester, Chester, United Kingdom

**Keywords:** SORT IT, Operational research, length of hospital stay, institutional deliveries, malnutrition

## Abstract

**Introduction:** Out-of-pocket fees to pay for health care prevent poor people from accessing health care and drives millions into poverty every year. This obstructs progress toward the World Health Organization goal of universal health care. Community-based health insurance (CBHI) improves access to health care primarily by reducing the financial risk. The association of CBHI with reduced under-5 mortality was apparent in some voluntary schemes. This study evaluated the impact of eQuality Health Bwindi CBHI scheme on health care utilization and under-5 mortality in rural south-western Uganda.

**Methods:** This was a retrospective cross-sectional study using routine electronic data on health insurance status, health care utilization, place of birth, and deaths for children aged under-5 in the catchment area of Bwindi Community Hospital, Uganda between January 2015 and June 2017. Data was extracted from four electronic databases and cross matched. To assess the association with health insurance, we measured the difference between those with and without insurance; in terms of being born in a health facility, outpatient attendance, inpatient admissions, length of stay and mortality. Associations were assessed by Chi-Square tests with *p*-values < 0.05 and 95% confidence intervals. For variables found to be significant at this level, multivariable logistic regression was done to control for possible confounders.

**Results:** Of the 16,464 children aged under-5 evaluated between January 2015 and June 2017, 10% were insured all of the time 19% were insured for part of the period, and 71% were never insured. Ever having had health insurance reduced the risk of death by 36% [aOR; 0.64, *p* = 0.009]. While children were insured, they visited outpatients ten times more, and were four times more likely to be admitted. If admitted, they had a significantly shorter length of stay. If mother was uninsured, children were less likely to be born in a health facility [adjusted odds ratio (aOR) 2.82, *p* < 0.001].

**Conclusion:** This study demonstrated that voluntary CBHI increased health care utilization and reduced mortality for children under-5. But the scheme required appreciable outside subsidy, which limits its wider application and replicability. While CBHIs can contribute to progress toward Universal Health Care they cannot always be afforded.

## Introduction

Most people across the world find it difficult to pay out-of-pocket (OOP) fees to access health services and this has become a barrier for the millions of poor people resulting in increased morbidity and mortality (1, 2). High income countries have developed ways of funding health care through taxation or compulsory insurance over the last fifty years or more, but most low and middle-income countries have a small tax base and cannot afford to adequately fund health care services (3). Health insurance can assist people who cannot afford the risk of OOP fees for health care (4).

Health insurance increases utilization of primary care for its beneficiaries. Research from USA and South Korea shows that insured children are more likely to visit physicians and dentists, use preventive care, utilize outpatient and emergency services and use inpatient health care (5–9). Uninsured children are more likely to have impaired access to specialties, and increased utilization of emergency departments, have avoidable hospitalizations and admissions, and are more likely to die in hospitals (10–12).

The World Health Organization (WHO) recommends Community Based Health Insurance (CBHI) as one of the approaches for reducing catastrophic out-of-pocket expenditure for registered families. It is promoted as one of three ways of achieving universal access to health care. The other two are social insurance and taxation, both of which require a large formal economy (13, 14). The 58th World Health Assembly focused on health care financing in order to promote access sharing risk and protecting poor people from impoverishment due to catastrophic health-care expenditures (15, 16).

CBHI was developed in late 1980s as a way of funding health care in low income countries and encouraging equity (17). It is now a way that some governments fund health care, particularly in low and middle-income countries. CBHIs increase financial protection and mobilize revenues to supplement health care funding and has been found beneficial for poorer communities that struggle to afford OOP. CBHI is based on a resource pooling approach through organized community structures, social groups or families with an aim of improving health (18–20). How well it achieves this is debated (21, 22).

CBHIs in Africa do not have high enrollment, having less than 10% of the targeted population insured, most for <10 years. Rwanda is an exception to this with an enrollment of over 80% of the population, but their scheme no longer meets the definition of CBHI as it has been compulsory since 2006 (23). Factors that affect enrollment include education status, lack of trust in health service providers, unclear regulatory framework for the CBHIs, enrollment requirements, managerial capacity, and previous health utilization history of households. CBHIs require substantial investment to start, and can be misunderstood by the informal sector (mainly the uneducated population), but may also lack political commitment (24–31).

In countries where CBHIs have been introduced, such as Rwanda, Senegal, and the Philippines, positive health outcomes have been observed for CBHI subscribers. Studies indicate a higher utilization of health care among children under-5, reduction in catastrophic health expenditure, improvement of health indicators such as completion of immunization, and reduction in under-5 mortality for the insured (18, 18, 32–34). Some studies show CBHI associated with increased outpatient attendance but no significant increase in inpatient admission, but did not look specifically at children under-5 (35–37). There is also an increase in institutional deliveries (19, 32, 38).

## Background and rationale

Health care in Uganda is provided by government health facilities, which provide free treatment and investigations, but frequently have limited supplies and staff. Private not-for-profit facilities, private for-profit facilities and NGOs, have more adequate supplies and staffing, however are expensive. These institutions are typically supported by development partners (39)^1^. According to the Ministry of Health, Uganda 2016, public health financing represents only 17% of the total health expenditure whereas health development partners and the private sector account for 41 and 42% respectively (40).

Paying out-of-pocket health care fees is a challenge to the poor who cannot afford to pay for health care. Uganda removed health care user fees in 2003 which increased utilization of health care, but catastrophic expenditure did not fall, due to the infrequent availability of drugs in government facilities. There are limited prepayment mechanisms for health care in Uganda (41, 42).

Health insurance, and more particularly CBHI, is not an approach that has been widely used for health care financing in Uganda. CBHIs began in 1996 in Uganda and have spread slowly (13). Most of these schemes are administered by faith-based hospitals. The schemes are usually started with the knowledge of the Ministry of Health and are supported by donors. Enrollment is limited to small geographic areas and remains at only two percent (16, 41–43). There is continued interest in expanding CBHIs but they require a careful design (23).

Bwindi Community Hospital (BCH) is a private not-for-profit community hospital operated under the auspices of the Church of Uganda. It is in Kigezi region in south western Uganda. BCH is located at the edge of Bwindi Impenetrable National Park, close to the Ugandan boarder with the Democratic Republic of Congo. Because of its mountainous nature and poor road network, this region is classified by the Ugandan Ministry of Health as “hard to reach” (poor transport links) and “hard to stay” (high staff turnover). There is a lack of public transportation and there are relatively few cars, buses, motorcycles and there are no sealed roads in the district. BCH serves some of the poorest and most remote populations in Uganda.

BCH's catchment consists mainly of three sub-counties; Kayonza, Kanyantorogo, and Mpungu, comprises 101 villages and an estimated population of 71,000. 12,000 (17%) are aged under-5.

According to BCH's 2016/2017 annual report (44), the under-5 mortality in their catchment area [51 per 1,000 live births (0.51%)] is lower than the national rate (64 per 1,000 live births (0.64%). In Uganda, the main causes of death in this age group are pneumonia, malaria, and diarrheal disease and HIV/AIDS^1^.

eQuality Health Bwindi (eHB) is a community-based health insurance scheme started by BCH in 2010 to enable people who reside in its catchment area to access health care with less financial risk. eHB is provided at BCH and at two outlying health centers run by the hospital. These three institutions are the participating health facilities. There are government health centers in the district but they do not participate in eHB.

Mothers and children under five often die of diseases that can easily be treated, but accessing treatment may be delayed if there is a risk of appreciable financial cost and if it is physically harder to reach. It was expected that eHB might increase outpatient attendance and admission to some extent and might reduce under-five mortality. It was thought that distance from participating health care facilities, and living in a hard to reach area might affect being born in a health unit, insurance uptake, and access to health care, but it was not known if this would be significant.

### Study population

The study population was all children aged under-5 residing in the 3 Sub Counties of Kigezi Region of Uganda between January 2015 and June 2017 who were registered on the Community Health Electronic Database.

The eHB scheme removes many of the economic challenges for families and their children to access health care. However, the scheme has not been evaluated to determine the difference in outcomes or health care utilization between those children with and without insurance. This study aimed to compare children under-5 with and without health insurance to ascertain if it affected the use of the participating health facility use and/or under-5 mortality rates in the catchment area of BCH, Uganda between January 2015 and June 2017.

The objectives were to assess the following for children with and without insurance

- The demographic and social characteristics of the children,
- Factors related to the number and percentage of children born in a participating health facility,
- The number and percentage of Outpatient and Inpatient attendances, and related factors including length of admission, and
- Factors related to the numbers and rate of under-5 deaths.

## Description of the case

The eHB Scheme had 30,000 registered members in 2017. It was organized at a community level. Participation was predicated upon a requirement that families be a member of an accepted social group (a community recognized organized group of people or households with common goals of which one is promotion of health). For members of such group to be able to subscribe to eHB, at least 60% of the group had to be willing to join and to pay subscription fees.

This health insurance resulted in subscribers accessing health care at a much-reduced cost when they were ill. eHB Scheme cost USD $5 per year for an adult and $3 for a child. The annual payment was paid in 3 equal portions over the year. The average annual rural income in Uganda was about $575, but many live on much less (45). Some families were not able to afford to have continuous health insurance, thereby risking a catastrophic health expenditure.

Adults with insurance paid $0.66 instead of $6.66 for an outpatient attendance. Children paid half of this ($0.33). The reduced outpatient fee for those insured also covered investigations and treatment, while the uninsured paid for each test and treatment. Those with insurance paid only 20% of the cost of surgical procedures, the other costs of admission were offered at a reduced charge.

## Methods

The data were extracted from four routinely maintained electronic databases; the community health database (data on household members' demographic details), eHB database (data on demographic and subscription status details of household's members who were currently or previously registered as insured), and the outpatient and inpatient databases. Since 2012, BCH has been collecting data electronically from the community and the hospital.

Cross matching the data was challenging in terms of correctly identifying individuals and families on each database. Extra validation was undertaken to reconcile data by visiting villages to confirm details entered on the databases. Efforts were made to cross-match every child under-5 and household against registered house information. This resulted in the identification of the insurance status of 97% of all children under-5 known to the BCH Community Health Services.

### Study design

This study was a retrospective cross-sectional study using routinely recorded data.

### Study setting

Uganda is a landlocked country in East Africa with a population of 34.6 million. The population growth rate is 3% per year and 48% of general population is aged under 14 years. Eighty percent of the population are subsistence farmers and 78% of the population live in rural areas. (36) The area served by eHB is remote and hard to reach with majority of the population classified as very poor.

### Data variables and sources

The independent data variables collected were insurance status, gender, residence in “hard to reach area” (an area on a steep ridge over 500 m high, only accessible on foot) or not, and distance from a participating health facility. The output variables included under-5 mortality, outpatient attendance, inpatient admission and length of stay, and place of birth, nutrition and immunization status.

Because a household's insurance status was something that could change from month to month, four different variables representing insurance status were derived from the households' insurance history data, in order to provide the most meaningful variable to compare with each of the different output variables. These were:

- The insurance status of the mother of the household at the time of the child's birth (for comparison with place of birth);
- The overall number of children under 5 in households who were insured some, all, or none of the time
- The insurance status of the child/child's household when the child attended or was admitted to a participating health unit
- The percentage of months of a child's life under 5 years in the study period for which the household was insured (for comparison with whether the child died, whether the child reached full immunization status, and the poorest nutritional status recorded for the child during the study period).

The sources of data were the eHB database, the Community Health Database, outpatient and inpatient databases.

## Analysis and statistics

Data was exported from four electronic databases and cross matched. The reconciled data imported into Epidata and analyzed using Epidata (version 2.2.2.186, EpiData Association, Odense, Denmark). The variables were then summarized using tables and graphs.

To assess the impact of CBHIs on under-5 mortality, outpatient attendance, inpatient admissions and place of births, the Chi-Square test was used and results presented as odds ratios and *p*-values at 95% confidence intervals (CIs). For variables found to be significant at this level, multivariable logistic regression was done to control for possible confounders. Levels of significance were set at *P* < 0.05.

### Ethics approval

Ethical Approval for the study was obtained from the BCH Health and Scientific Committee, and from The Union Ethics Advisory Group, Paris, France.

## Results

Of the 16,464 children who were aged under-5 (Table 1), (11,712, 71%) were never insured, (3,103, 19%) were insured for part of the period and only (1,641, 10%) were insured all the time. This means (4,744, 29%) were insured for some or all the period.

**Table 1:** Study population by the study's independent variables.

**Variable**	**Number of births during period**	**Number of children under-5 during period**	**Number of months of under-5 child's life during period**
All	5,109	16,464	343,918
**GENDER**			
Male	2,580 (50%)	8,274 (50%)	172,546 (50%)
Female	2,529 (50%)	8,190 (50%)	171,372 (50%)
**INSURANCE STATUS**			
Not insured	3,852 (77%)	11,712 (71%)	280,642 (82%)
Insured	1,257 (23%)	4,744 (29%)	63,276 (18%)
**of which insured for:**
1–49% of months	n/a	1,729 (11%)	n/a
50–99% of months	n/a	1,374 (8%)	n/a
100% of months	n/a	1,641 (10%)	n/a
Not recorded		8 (0%)	
**VILLAGE DISTANCE FROM PARTICIPATING HEALTH FACILITY**			
Near (<5 km)	1,475 (29%)	4,836 (29%)	102,562 (30%)
Medium (6–15 km)	1,231 (24%)	3,947 (24%)	83,121 (24%)
Far (16–20 km)	869 (17%)	2,840 (17%)	59,929 (17%)
Very Far (>20 km)	1,224 (24%)	3,908 (24%)	79,251 (23%)
Not Recorded	310 (6%)	933 (6%)	19,055 (6%)
RESIDENT IN A HARD TO REACH AREA^*^			
Not hard-to-reach	3,451 (68%)	11,172 (68%)	233,400 (68%)
Hard-to-reach	1,658 (32%)	5,272 (32%)	110,518 (32%)

Numbers of (a) children under-5, b) births, and (c) months of under-5 child's life, during January 2015 to June 2017 in the catchment of Bwindi Hospital Uganda, by the study's independent variables: gender; the child's insurance status; distance of home village from a participating health facility; and whether resident in a hard to reach area

* *A “hard to reach” area is on a steep ridge, only accessible on foot, not accessible by motorbike*.

*“Participating health facility”—facilities where care is provided under the eHB scheme*.

Children were 2.4 times more likely not to be born in a health facility (Table 2) if the mother was not insured, and 1.8 times more likely not to be born in a health facility if they lived in a hard to reach area. The results also indicated that children were more likely to be born in a health facility if they lived more than 5 km from a health facility participating in eHB. This will be influenced by a number of factors, including that there are other health facilities in the area besides the eHB-participating ones where children can be delivered, and that the eHB facilities are located quite close in pure distance terms to some of the more hard to reach villages.

**Table 2:** Factors associated with a child being born in a participating health facility in the catchment of Bwindi Community Hospital, Uganda, between January 2015 and June 2017.

**Variable**	**Health Facility delivery n (%)**	**Home delivery n (%)**	**OR (*P*-value–chi-square)**	**aOR (*P*-value–logistic regression)**
**INSURANCE STATUS AT BIRTH**				
Mother insured	1,186 (94.4)	71 (5.6)	1	
Mother not insured	3,360 (87.2)	492 (12.8)	2.44 (<0.001^*^)	2.82 (<0.001^*^)
**RESIDENT OF A HARD TO REACH AREA**^+^ **OR NOT**				
Yes	1,408 (84.9)	250 (15.1)	1	
No	3,138 (90.9)	313 (9.1)	1.78 (<0.001^*^)	1.47 (<0.001^*^)
**DISTANCE FROM NEAREST PARTICIPATING HEALTH FACILITY**^#^				
Near (<5 km)	1,241 (84.1)	234 (15.9)	1	
Medium (6–15 km)	1,096 (89.0)	135 (11.0)	0.65 (<0.001^*^)	0.63 (<0.001^*^)
Far (16–20 km)	786 (90.4)	83 (9.6)	0.56 (<0.001^*^)	0.52 (<0.001^*^)
Very Far (>20 km)	1,153 (94.2)	71 (5.8)	0.33 (<0.001^*^)	0.44 (<0.001^*^)
Not Recorded	270 (87.1)	40 (12.9)	0.79	-

* *statistically significant at the 0.05 level*.

+ *A “hard to reach” area is on a steep ridge, only accessible on foot, not accessible by motorbike*.

# *“Participating health facility” – facilities where care is provided under the eHB scheme*.

There were more than twice as many outpatient attendances at the hospital by children who were insured at the time of attendance than by those who were not, despite a much smaller proportion of children being insured (Table 3). To take account of the fact that some children were only insured part of the time, the number of outpatient attendances and admissions per month-insured was compared with per month-uninsured. Those insured attended more, by a factor of ten; 0.123 per insured month compared to 0.012 per uninsured month. Those living in a hard to reach area were less than half as likely to attend out patients and number of attendances reduced rapidly with distance. Those living 6–15 k away attended about a third as often and those living 15–20 k away attending only 12% as often.

**Table 3:** Factors associated with children under-5 (a) Outpatient attendances and (b) Inpatient admissions at Bwindi Community Hospital, Uganda, between January 2015 and June 2017.

**Variable**	**Number of attendances/admissions**	**Number of months of under-5 child's life during period**	**Number of attendances/admissions per month of under-5 child's life**	**OR (*P*-Value-chi-square)**	**aOR (*P*-Value–logistic regression)**
**a) OUTPATIENT ATTENDANCES**					
**Insurance status**					
Not insured	3,411	280,642	0.012	1	
Insured	7,761	63,276	0.123	11.3 (<0.001)	8.13 (<0.001)
**Resident of a hard to reach area**^+^ **or not**					
Not hard-to-reach	9,340	233,400	0.040	1	
Hard-to-reach	1,826	110,518	0.017	0.40 (<0.001)	0.54 (<0.001)
Not classified	6	0	-	-	
**Distance from nearest participating health facility**^#^					
Near (<5 km)	7,989	102,562	0.078	1	
Medium (6–15 km)	2,217	83,121	0.027	0.32 (<0.001)	0.38 (<0.001)
Far (16–20 km)	535	59,929	0.009	0.10 (<0.001)	0.17 (<0.001)
Very far (>20 km)	179	79,251	0.002	0.03 (<0.001)	0.05 (<0.001)
Not recorded	251	19,055	0.013	0.16 (<0.001)	0.15 (<0.001)
**b) INPATIENT ADMISSIONS:**					
**Insurance status**
Not insured	1,003	280,642	0.004	1	
Insured	848	63,276	0.013	3.79 (<0.001)	3.81 (<0.001)
**Resident of a hard to reach area**^+^ **or not**					
Not hard-to-reach	1,533	233,400	0.007	1	
Hard-to-reach	318	110,518	0.003	0.44 (<0.001)	0.52 (<0.001)
Not classified	0	0	–	–	
**Distance from nearest participating health facility**^#^					
Near (<5 km)	1,177	102,562	0.011	1	
Medium (6–15 km)	369	83,121	0.004	0.39 (<0.001)	0.46 (<0.001)
Far (16–20 km)	146	59,929	0.002	0.21 (<0.001)	0.29 (<0.001)
Very far (>20 km)	72	79,251	0.001	0.08 (<0.001)	0.11 (<0.001)
Not Recorded	87	19,055	0.005	0.40 (<0.001)	0.23 (<0.001)

+ A “hard to reach” area is on a steep ridge, only accessible on foot, not accessible by motorbike.

# *“Participating health facility”—facilities where care is provided under the eHB scheme*.

Children who were insured were admitted more, by a factor of four (0.013 compared to 0.004). Those from hard to reach areas were admitted less than half as often and again the proportion of children admitted reduced rapidly with distance from a participating health facility.

For children admitted to hospital, those with insurance had a significantly shorter length of stay (median 2 days, interquartile range 1 day to 5 days) than those without insurance, (median 10 days interquartile range 3 days to 18 days), *p* < 0.02 Mann Whitney U test.

The proportion of children aged more than 9 months who had a complete immunization status was over 91% for all children and was not affected by insurance status or living in a hard to reach area Table 4. The pattern of immunization completeness relative to distance from a participating health facility was less clear.

**Table 4:** Factors associated with (a) whether a child aged 9 months or more at the end of the period was recorded as having reached full immunization status, and (b) whether a child aged 6 months or more at the end of the period was identified with malnutrition around Bwindi Community Hospital, Uganda, during the period January 2015 and June 2017.

**Variable**	**Yes *n* (%)**	**No *n* (%)**	**OR (*P*-value–chi-square)**	**aOR (*P*-value–logistic regression)**
**a) WHETHER REACHED FULL 9 MONTH IMMUNISATION STATUS IN PERIOD:**				
**Insurance status**				
Never insured	9,886 (94.1%)	616 (5.9%)	1.04 (0.59)	–
Partly or fully insured	4,177 (93.9%)	271 (6.1%)	1	
Not recorded	4 (50%)	4 (50%)	–	
**Gender**				
Female	7,004 (94.0%)	451 (6.0%)	1	
Male	7,063 (94.1%)	440 (5.9%)	1.03 (0.63)	–
**Resident of a hard to reach area**^+^ **or not**				
No	9,547 (94.3%)	581 (5.7%)	1	
Yes	4,520 (93.6%)	310 (6.4%)	0.89 (0.09)	-
**Village distance from nearest participating health facility***^#^*				
Near (<5 km)	4,131 (92.8%)	319 (7.2%)	1	1
Medium (6–15 km)	3,263 (91.1%)	318 (8.9%)	0.79 (0.005)	0.79 (0.005)
Far (16–20 km)	2,443 (94.6%)	140 (5.4%)	1.35 (0.004)	1.35 (0.004)
Very Far (>20 km)	3,441 (97.9%)	76 (2.1%)	3.50 (<0.001)	3.50 (<0.001)
Not recorded	789 (95.4%)	38 (4.6%)	1.60 (0.007)	1.60 (0.007)
**b) WHETHER IDENTIFIED WITH EITHER MILD OR SEVERE MALNUTRITION IN PERIOD:**				
**Insurance status**
Never insured	171 (1.8%)	9,442 (98.2%)	1	
Partly or fully insured	52 (1.3%)	4,044 (98.7%)	0.71 (0.03)	0.63 (0.004)
Not recorded	0 (0%)	7 (100%)	–	
**Gender**				
Female	106 (1.6%)	6,716 (98.4%)	0.92 (0.55)	–
Male	116 (1.7%)	6,777 (98.3%)	1	
**Resident of a hard to reach area**^+^ **or not**				
No	137 (1.5%)	9,130 (98.5%)	0.77 (0.61)	–
Yes	85 (1.9%)	4,363 (98.1%)	1	
**Village distance from nearest participating health facility***^#^*				
Near (<5 km)	98 (2.4%)	3,979 (97.6%)	1	
Medium (6–15 km)	35 (1.1%)	3,279 (98.9%)	0.43 (<0.001)	0.42 (<0.001)
Far (16–20 km)	35 (1.5%)	2,326 (98.5%)	0.61 (0.013)	0.56 (0.004)
Very Far (>20 km)	40 (1.2%)	3,163 (98.8%)	0.51 (<0.001)	0.45 (<0.001)
Not recorded	14 (1.8%)	744 (98.2%)	0.76 (0.35)	0.71 (0.24)

+ A “hard to reach” area is on a steep ridge, only accessible on foot, not accessible by motorbike.

# *“Participating health facility” – facilities where care is provided under the eHB scheme*.

Children who had ever been insured were at less risk of malnutrition (OR 0.71; *p* = 0.03) compared to the never insured (Table 5). The results also showed that children were at less risk of malnutrition if they lived more than 5 km from a health facility participating in eHB.

**Table 5:** Factors associated with a death under-5 in the catchment of Bwindi Community Hospital Uganda, between January 2015 and June 2017.

**Variable**	**Died *n* (%)**	**Survived *n* (%)**	**OR**	**Aor (*P*-value-logistic regression)**
**Insurance status**				
Never insured	203 (1.7%)	11,509 (98.3%)	1	
Partly or fully insured	49 (1.0%)*	4,695 (99.0%)	0.59	0.64 (0.009^*^)
Not recorded	6 (75%)	2 (25%)	170	
**Gender**				
Female	101 (1.2%)	8,089 (98.8%)	1	
Male	157 (1.9%)	8,117 (98.1%)	1.55	1.6 (<0.001)
**Resident of a hard to reach area**^+^ **or not**				
No	156 (1.4%)	11,016 (98.6%)	1	
Yes	102 (1.9%)	5,190 (98.1%)	1.38	1.37 (0.02)
**Village distance from nearest participating health facility***^#^*				
Near (<5 km)	66 (1.4%)	4,770 (98.6%)	1	
Medium (6–15 km)	57 (1.4%)	3,890 (98.6%)	1.06	1.02 (0.94)
Far (16–20 km)	48 (1.7%)	2,792 (98.3%)	1.24	1.16 (0.45)
Very Far (>20 km)	78 (2.0%)	3,830 (98.0%)	1.47	0.89 (0.88)
Not recorded	9 (1.0%)	924 (99.0%)	0.7	

* Combined due to small numbers.

^+^A “hard to reach” area is on a steep ridge, only accessible on foot, not accessible by motorbike.

# *“Participating health facility” – facilities where care is provided under the eHB scheme*.

Ever having had health insurance reduces the risk of dying under-5 by 36% (Table 5). Living in a hard to reach area increased the risk of dying (aOR 1.4 *p* = 0.02), but distance from the health facility did not.

## Discussion

This is the first study that demonstrates that insured under-5s admitted to hospital have a shorter length of stay than those without. We also found that under-5s who were insured were more likely to be born in a health facility, attended outpatients department ten times as often, were more likely to be admitted to hospital and experienced a lower mortality rate compared to the uninsured.

The risk of death in the under-5 age was 36% less in the insured versus non-insured. This is comparable with findings from the Burkino Faso study (34) that discovered a 46% reduction in risk of death in children who had ever had health insurance. In Rwanda, under-five child mortality and infant mortality reduced tremendously with introduction of CBHI. However, due to insufficient data at the time this study was conducted it was not possible to measure the impact of CBHI (*Mutuelles)* under-5 mortality (18).

The increase in health care utilization by those with insurance was much higher by factor of 10 compared with a factor of 1.4 in the Burkino Faso study. The reasons for this are not known. Other studies in Burkina Faso and Ethiopia have shown an increase in all outpatient attendances, not specifically in the under-5s. In Burkina Faso CBHI insurance increased outpatient attendances by 40%, whereas in Ethiopia the insured had 0.19 visits compared to 0.11 among the insured (35, 36). Unlike in this study, these studies in Burkina Faso and Ethiopia do not provide evidence that CBHI increase inpatient attendance. In Senegal, it was noted that CBHI membership has a moderate positive effect on the probability of going to a hospital of two percent when the insured were ill (30). In Rwanda, CBHIs introduction resulted in increased medical care utilization (including inpatient care, outpatient consultation, and medical tests and examinations) for under-fives with acute respiratory illness from 13% of children attending in 2000 to 33% in 2008 for selected common childhood illnesses (18).

The shorter length of hospital stay for those with insurance may reflect the fact that these children were admitted before they became severely ill, which may also have contributed to the reduction of under-5 mortality. Shorter length of stay may reduce the risk of households of admitted clients from experiencing catastrophic health expenditure that could result from out of pocket expenditure for the charges associated with the admissions. No other studies were identified that assess impact of CBHI on length of stay among children under-5. One study in Kenya that assessed this parameter in the general population indicated that the insured were significantly more likely to spend more days in hospital compared to the non-insured members (37). Reasons for this are not indicated in the study.

The possibility that children without health insurance were less likely to be born in a health facility may be associated with the fact that hospital-based deliveries are conducted at cost and those without health insurance may not be able to afford this cost. Although the cost is considerably lower in the participating health facilities, it might be quite expensive for this population. Studies in Rwanda, Kenya, and Ghana have showed similar results. In Rwanda, after introduction of CBHI, the utilization of skilled-birth attendants rose from 39% of births in 2000 to 67% in 2008 (18). In Kenya, 33.4% of the insured were hospitalized for child delivery in comparison to 26.7% of non-insured members (37). An increase in utilization of maternal health services by those with insurance has also been noted in a study conducted in three countries of Senegal, Mali, and Ghana (38).

A number of factors will affect the pattern of immunization completeness relative to distance from a participating health facility, including that immunization services are provided, both by the hospital and others, at a number of different locations in the area besides the eHB-participating health facilities.

Similarly, the counter-intuitive finding that children living more than 5 km from a health facility participating in eHPB were at less risk of malnutrition, will be influenced by a number of factors. Perhaps the most important is that the eHB facilities are located quite close in pure distance terms to some of the more hard to reach villages.

### Strengths of the study

We used data combined from the eHB and community health database to give comprehensive population-based data. The data were validated and cross matched against household information and we obtained the insurance status on 97% of the under-5s. Attendance and admission data was accessed for both those who were insured and who were not insured.

### Limitations

We were not able to identify outpatient re-attendances for uninsured children, and so do not know whether the patterns of re-attendance differed between insured and uninsured children. There may be other important factors that affect the outputs in this study which have not been routinely recorded. Insurance status could not simply be categorized as insured or not because 19% were insured for part of the time period, making it more difficult to assess the impact of insurance on some of the outcomes. There was little data on the costs of the eBH scheme and the level of subsidization available. The results of this study should be generalized with care, as the organization of the insurance scheme and local geography are unusual, and as with any case study, only an association, rather than causation, can be shown.

It is understandable that those living at greater distance from a health facility or in a hard to reach area are less likely to pay for health insurance, as access is more problematic. The higher mortality of children living in hard-to-reach areas, which cannot even be accessed by motorbike, showed the importance of reasonable access to health care.

## Lessons learned

This study confirms that under-5s with insurance have increased utilization of out-patient departments and experience lower mortality rates than those without insurance, even in a small to medium size CBHI scheme. It shows that under-5s with insurance are more likely to be admitted to hospital and if admitted, to have a shorter hospital stay.

It appears that being covered by health insurance even for part of the time helps to reduce mortality. It may be that with post-neonatal deaths some of the inputs related to the insurance, such as health education when attending hospital, may have longer term impact, or families who buy any health insurance are more aware of the importance of health and health care.

BCH has developed an approach to eHB to enable the community to become used to the concept health insurance through upfront payments via premiums and community cost-sharing. Currently the program is subsidized from outside the country and it was estimated to be about two thirds of the cost. More work needs to be done on this. As the communities become more familiar with this approach it is planned that the premiums and fees will gradually be increased, as has already been done over the past 4 years, to cover a larger share of the costs. The Ugandan government is considering a national insurance scheme, but details have yet to emerge. It should consider assuming responsibility for the continued funding of eHB.

The need for subsidization limits the expansion and replicability of CBHI. Voluntary CBHI schemes have not had the wide-spread uptake by local people to have a large impact on the health of an area, and eBH is no different. For these reasons there is a need to find a more sustainable funding model before eBH can be expanded locally.

## Conclusion

This study shows that under-5s with insurance are more likely to be admitted to hospital and if admitted, to have a shorter hospital stay. It confirms that under-5s with insurance have increased utilization of out-patient departments and experience lower mortality rates than those without insurance, even in a small to medium size CBHI scheme. This confirms that a CBHI contributes to improved health in under-5s. CBHIs are a valid concept toward Universal Health Care, but, at present, they are unlikely to be self-funding, requiring outside subsidization. This limits their wider adoption.

## Data availability statement

The datasets for this manuscript are not publicly available because: they are part of a large data which is still being worked on. Requests to access the datasets should be directed to [Nahabwe Haven, hahotice@googlemail.com].

## Author contributions

NH, AD, and EW conceived the study. NH, AD, EW, KY, AS, and BM designed the study protocol and all authors read and approved the study protocol. NH, and AD collected the data. All authors contributed to analyzing and interpreting the data. NH, AD, EW, SK, and AS drafted the manuscript and all authors critically revised the manuscript for intellectual content. All authors read and approved the final manuscript. NH and AD are guarantors of the paper.

### Conflict of interest statement

The authors declare that the research was conducted in the absence of any commercial or financial relationships that could be construed as a potential conflict of interest.

## Footnotes

^1^http://www.who.int/health_financing/documents/cov-hsfr_e_10-uganda/en/
